# Biometric Identification Based on Keystroke Dynamics

**DOI:** 10.3390/s22093158

**Published:** 2022-04-20

**Authors:** Pawel Kasprowski, Zaneta Borowska, Katarzyna Harezlak

**Affiliations:** Departament of Applied Informatics, Silesian University of Technology, Akademicka 16, 44-100 Gliwice, Poland; zanebor793@student.polsl.pl (Z.B.); katarzyna.harezlak@polsl.pl (K.H.)

**Keywords:** neural network, biometric identification, keystroke dynamics

## Abstract

The purpose of the paper is to study how changes in neural network architecture and its hyperparameters affect the results of biometric identification based on keystroke dynamics. The publicly available dataset of keystrokes was used, and the models with different parameters were trained using this data. Various neural network layers—convolutional, recurrent, and dense—in different configurations were employed together with pooling and dropout layers. The results were compared with the state-of-the-art model using the same dataset. The results varied, with the best-achieved accuracy equal to 82% for the identification (1 of 20) task.

## 1. Introduction

Biometrics is a branch of science that focuses on quantitative research on the population and its variability based on measurements of living beings’ characteristics. Such investigations aim to label and describe individuals and can be utilized for subjects’ verification or identification to protect various resources. The verification process begins with the user declaring their identity. The feature used in a given system is compared with that previously registered. If the verification is positive, the user can access the requested resources. Identification is the process of determining the identity without any declaration [1]. The measured feature is compared with the previously taken measurements. Due to the greater complexity of the task (1:n), identification may be considered more complex and more time and resource consuming.

Human features currently known and used in biometrics are based on physiological and behavioral characteristics. In the first group, the fingerprint, iris pattern of the eye, hand geometry, vascular structures and ear shape can be listed. The second group includes such characteristics as way of walking, handwritten signature, eye movement, and voice [2].

Although biometric systems have been known for many years, the increased interest began in the 1990s and continues to this day. It stems from the fact that the range of areas for the present and future use of biometrics is wide [1], as for example internet transactions, workplaces, access to corporate networks or resources, telephone transactions, as well as travel and tourism. The main advantages of biometrics include:

- No need to remember passwords or keep other items/tokens allowing access to resources;
- Increased security. The use of biometric data allows protecting against some counterfeit attacks (including phishing);
- Impossible to be forgotten or lost.

Currently, the most frequently used biometric system is fingerprint scanning. Fingerprints are used on a daily basis, including forensics science (identification and verification), as confirmation of the authenticity of documents (e.g., in notarial deeds, in the case of patients), or in smartphones. Biometric systems that use facial analysis are also becoming more and more popular. Their advantages include, among others, no need to use specialized equipment—all that is needed is a camera. The above systems can also be used without the awareness of the person whose face is being analyzed—which, apart from the privacy aspect, can be successfully used in places requiring increased security control, such as airports.

Some solutions use more than one feature. An example may be fingerprint scanners, which also apply such parameters as temperature, capacity, humidity, or the distribution of veins in the finger. There have also been attempts to use the so-called soft biometrics to speed up the action and increase the overall performance [3]. Face size, gender, skin color, ethnicity, and height can be mentioned among them.

Behavioral biometrics is undoubtedly used much less frequently. Interest in this branch appears more often in scientific works than in commercial applications. The most popular use of behavioral traits is voice biometrics, which is used by some financial institutions [2] and in e-commerce. Additionally, language, accent, speed and way of speaking, the pitch of voice, its frequency, nasal sound, and intonation are some of the features taken into account. In voice identification and/or authentication, analysis of frequency vibrations of the voice waves is increasingly used. For this purpose, the Fourier transform is applied, and the obtained spectrum is compared using various algorithms, including those using artificial neural networks. The disadvantage of voice biometrics is undoubtedly the influence of acoustic, emotional, or health conditions on the result of the verification of an individual. A relatively new group of behavioral features includes those obtained through interaction with a computer system: not only the dynamics of typing, which is the subject of this work, but also the dynamics of computer mouse movements or interaction with the software interface. Each of the above-mentioned features is the subject of many studies, but also there are works applying several of them simultaneously [4].

Despite increasing popularity, biometric systems are not perfect. Studies have shown that even the iris of the eye, considered to be the most perfect human identifier, is not resistant to aging, and in just 3 years, its structure may change so that the biometric system will no longer recognize it [5]. Both physical and biometric characteristics can also change as a result of disease or accidents. The disadvantage may also be the need to use specialized, sometimes expensive equipment, or low-quality devices, which may give a deceptive sense of security.

The physical or behavioral features have to meet some conditions to apply them for personal identification. They should be characterized by:

- Uniqueness—they should not repeat among people;
- Universality—they should be common among individuals;
- Ease of collection—it should be feasible to register a sample easily, quickly, and non-invasively;
- Stability (immutability)—the biometric source should be (relatively) unchanged over time;
- Acceptability (among users)—the use and collection of a given feature should not raise any objections; it should be friendly and convenient.

Biometric systems usually are based on two steps [6]. The first one is to collect the user’s biometric data and typically consists of sample capturing and feature extraction. The latter, saved, are further used to compare with samples obtained and processed in the same way during the second stage—identification or verification. Obtaining recordings identical to the template is almost impossible, which implies that biometric solutions will never give a perfect adjustment. For example, external factors or different equipment used may cause differences between the measurements.

Much research focused on biometric tasks applies machine learning methods, and neural networks are among them. Because the registered keystrokes constitute a time series, recurrent neural networks (RNNs), a class of neural networks (NN), seem naturally suited to processing such data. It is a type of NN, which is widely used to perform the sequence analysis process, as the RNN is designed for extracting the contextual information by defining the dependencies between various timestamps [7]. The use of the standard RNN is not recommended for long-term dependencies. For this reason, the LSTM (Long Short-Term Memory) network was invented. In the literature, GRU (Gated Recurrent Unit) recurrent networks can also be found, which is a modification of the LSTM network with fewer parameters. It results in fewer operations required to be performed, making the network faster to train. Additionally, there are studies utilizing convolutional neural networks (CNNs), although they are used primarily in image processing, classification, segmentation, as well as pattern detection and recognition processes. Nevertheless, they reveal good performance also in other classification tasks.

The research presented in this paper aims to examine different neural network architectures and their hyperparameters to find the best combination that gives the best identification accuracy. The publicly available dataset containing keystroke dynamics data recorded for many participants was used. In five subsequent experiments, various models with different combinations of layers were used and tested. There were different convolutional and recurrent networks utilized as well as the combinations of these types. It was also studied how additional layers, max-pooling, and dropout influence the results depending on their existence and hyperparameters. The main contribution of the paper is the detailed analysis of the performance of different models, including a model known from the literature.

## 2. The State of the Art in Keystroke Dynamics Identification

The first mention of the use of the typing method to identify an individual can be dated to the period of the Second World War. Back then, a common way of communication was to send messages using the Morse code, and operators quickly learned how to write correspondence. It was said that “the way the Morse code is sent is almost as distinctive as the voice”. This dependency proved helpful during the war, as it allowed distinguishing messages from allies and enemies, thanks to the different “ways” of typing [8]. Currently, the use of the Morse code is not that common anymore, but the same authentication method can be performed by pressing the keys on the keyboard. The approach seems promising; it is utilized as an additional solution, among others, on the Coursera platform [9]. It has been patented [10] and is used to authenticate users during the course assignments. In order to confirm their identity, students have to rewrite a specific sentence (the same as before the start of the course, which later serves as a model).

Many existing solutions are based on statistical data on the specific events, the number of their occurrences in time, or simultaneous occurrences of a chosen set of them [11]. The most frequently used characteristics include: the duration of a specific keypress and intervals between keypresses, typing speed (the average number of keystrokes in a given time), overlapping of a certain key combinations, ratio of Shift or Capslock buttons usage to type upper/lowercase letters, number of errors, error correction methods, and the use of the navigation (arrow) keys for the cursor. Only two features and a simple classifier can allow for quite effective authorization or identification of the user [11].

Pressing a key, understood as the time to insert a letter into the text, generates three primary time events: pressing the key (key down), releasing the key (key up), and pressing the key (keypress) [12]. The listed events are used to extract features divided into two groups: global and temporary ones. Global characteristics describe general user behavior while typing, such as error frequency, letter deletion, use of Shift, Control, Alt keys, general typing speed (keystrokes or words per minute). Temporary characteristics refer to the typing style of specific keys or combinations of keys. Based on them, the following features can be extracted.

- Interval—the time between the release of one key and pressing the next;
- Dwell time—the time between pressing and releasing the same key;
- Latency—the time between pressing one key and releasing the next one;
- Flight time—time between pressing one and the next key;
- Up to up—the time between releasing the first and next key.

The user’s identity verification is not the only typing dynamics application. It can also be utilized in users’ profiling. Over the past decade, keystroke-based pattern recognition techniques have gained increasing attention as a forensic tool in behavioral biometrics. The studies in this field have most often focused on determining the age and sex of an individual, but a machine learning model has also been created to assess the level of education [13]. Although the accuracy of such a model prediction was over 85%, this solution had several limitations, including high computational complexity, which is related to a long time of building the model.

Texts used in experiments may have a defined length (e.g., login or password) or be spontaneous, previously undefined content with an unknown number of letters. The first option is applied in most of the research concerning typing dynamics. Currently, works conducted in this scope focus on increasingly better processing of temporal features, the selection of appropriate algorithms, and their improvement [14,15]. The second approach with unknown, usually much longer, texts can be utilized for continuous authentication: for example, in the case of unprotected access to a system, when the user forgets to log out of it [12]. This strategy is being explored both toward the use of a standard keyboard and that of smartphones. In [16], the authors developed an Android application, iProfile, to collect keystroke events from Android devices. During the experiment, the participants typed a strong passcode. Based on the obtained characteristic, 155 features were defined, including dwell time, flight time, touch pressure, and their averages. The SVM classifier with two kernels, linear and RBF, was used for classification purposes. Two metrics were analyzed: F1 and accuracy. For accuracy, the RBF kernel gave 0.9740, while the linear one 0.9727. For the F1 score, the RBF kernel yielded a value of 0.9701, which was also greater than for linear: 0.9699.

Keyboard authentication using artificial neural networks is more and more frequently investigated. The solutions using convolutional [17], recursive [18] networks, as well as their combination [19] can also be found in the literature. For the above studies, the best EER values obtained are equal to: for CNN, 2.3% and 6.5% (with and without data augmentation); RNN, 13.6%; and CNN + RNN, 2.36% or 5.97%, depending on the dataset used.

In [20], the authors tested several deep neural network architectures for differentiating users based on their keystroke characteristics. They used two sets of keystroke features for this purpose. Two different feature sets are considered as well as networks with different numbers of hidden layers. The best results—a precision equal to 0.997 and a recall equal to 0.999—were obtained with an MLP network with nine hidden layers and the feature set composed by a higher number of features (including statistics of three consecutive key events).

The authors of the article [19] used a combination of convolutional and recursive networks. The research was conducted on two data sets: Clarkson II [21] and Buffalo [22]. The first of these was collected from 103 users in a completely uncontrolled natural environment over two and a half years, and users entered a total of 12.9 million keystrokes. The Buffalo dataset was gathered over three sessions in which users, at first, were asked to rewrite a given text and then answer the question in their own words. The study examined the influence of network parameters on the final result, i.e., sequence length, convolution kernel size, number of neurons in recursive networks, or the use of different configurations of the extracted features. The performance of the network for its various configurations was also compared. It revealed that the combination of the convolutional and recursive networks gave better results than the recursive network itself. In the case of the RNN, it was also found that the optimal solution is to use a simplified version of the LSTM, i.e., GRU.

Huang and co-authors, in their work [23], attempted to investigate the relationship between data size and authentication efficiency. Based on the conducted experiments, the researchers determined that with the increase of the sample size (the number of keystrokes), the error decreases. Additionally, they found out that to achieve good recognition and model reliability, it should contain at least 10,000 keystrokes and operate on approximately 1000 keystrokes.

Finally, in [24], the authors presented an LSTM-based network that was trained to convert keystroke data into embeddings the way that the embeddings collected for the same person are similar when the Euclidean distance between them is calculated. The method was successfully tested for a very big dataset containing data collected from thousands of users.

## 3. Materials and Methods

During the research, the Buffalo dataset was utilized. As mentioned, it consists of keystrokes registered while writing the text given by the organizers (the same for all respondents) and providing the answers to the questions (free text). The presented studies aimed to find a network model for undefined texts; therefore, a part of the set obtained while answering the questions was chosen. A single event in the dataset contained: the letter, information about whether it was pressed or released, and the event duration in milliseconds.

The Buffalo dataset consists of recordings obtained from 148 participants. Two subsets of this dataset were utilized in this research. One, consisting of 20 users, was used to optimize the architecture of the network in Experiments 1 to 4, and a bigger dataset of 54 users was applied during the comparisons presented in Experiment 5. Only open, free text, recordings were taken into account.

### 3.1. Data Preprocessing

**Figure 1:**
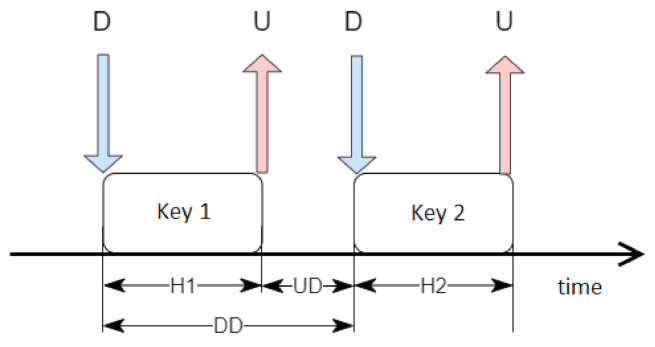
Time dependencies used.

Because the event duration, given in milliseconds, might not be sufficient to train the network correctly, some processing steps were required to prepare more comprehensive temporal characteristics, as shown in Figure 1. The letters D and U represent "down" and "up" and indicate the times of pressing and releasing the key, respectively. H1 and H2 are durations of holding two subsequent keys. They were calculated by the time difference between U and D. UD is the value of the time difference between pressing the next button and releasing the previous one. Finally, DD represents the period between pressing the key and the next one.

**Figure 2:**
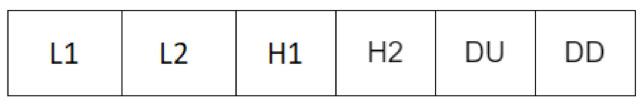
The structure of one vector of data.

At the end, the obtained vectors consisted of two consecutive letters (L1 and L2) and the described time characteristics. Their format is shown in Figure 2.

Due to the characteristics of the task (free text), recordings contained a different number of events. In order to minimize the impact of possible uneven data distribution and speed up the execution of the experiments, 1500 events from each recording were used for Experiments 1–4 and 2000 events from each recording were used for Experiment 5 (when the bigger dataset was analyzed).

The limitation of many machine learning algorithms is that they cannot run on categorical/text data. For this reason, it is required to convert the typed characters to numeric values. Due to the large number of characters used, simple conversion to successive numerical values could result in an incorrect learning process: poor performance or unexpected results. For this reason, the One Hot Encoding technique was used. It allows converting such values into binary variables. For each class, one column is assigned in the newly created vector. The swap puts the number one in place of the corresponding letter, while the rest of the vector is filled with zeros.

The next step was to standardize the data. This process was carried out separately for the time characteristics and letters transformed into binary vectors. For this purpose, the Z standardization was applied, which can be expressed by the following formula:

(1) $$z\;=\;\frac{x\;-\;\mu }{\sigma },$$

where:

- *x*—non-standardized variable;
- μ—the mean of the population;
- σ—standard deviation of the population.

**Figure 3:**
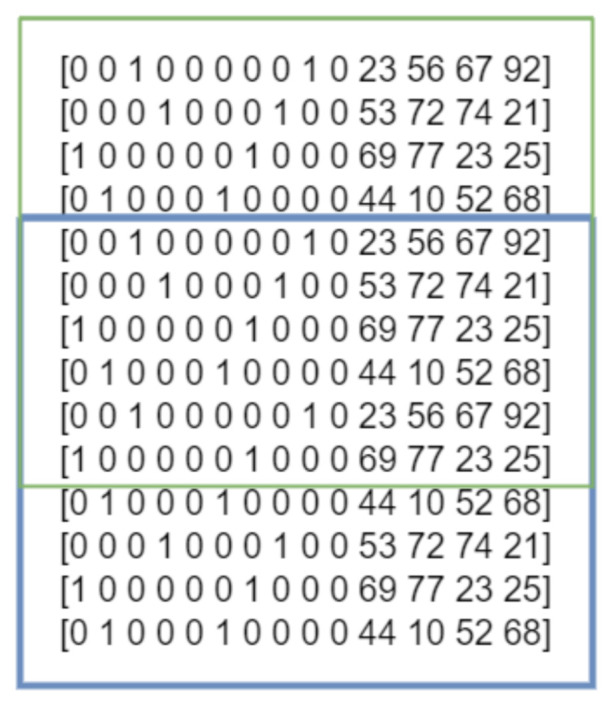
Sample with length equal to 10 with the shift of 40%.

The last stage of data processing was the creation of samples: two-dimensional arrays consisting of windows whose length was selected experimentally, which is discussed later in this work. Between two consecutive windows, there was a 40% shift in the window size, as shown in the Figure 3.

The obtained data were divided into training (75%) and test (25%) sets.

### 3.2. Experiments

In order to develop a suitable model for the identification process based on the typing style, various network configurations were analyzed. The results differed depending on the parameters studied:

- Window size;
- Kernel size;
- Number of filters in convolutional layers;
- Number of neurons in recursive layers;
- The type of recursive layer (LSTM or GRU);
- The value of the dropout rate parameter.

The desired goal of the experiment was to find a model that would assign the samples to the appropriate classes (users) with the greatest possible accuracy. For this reason, softmax was used as the activation function and the categorical cross-entropy was used as the loss function. The ADAM optimizer was used for all trials.

There were five experiments conducted:

1. In the first experiment, the model proposed in [19] was used. Four additional models were also created, being its modifications. All networks were trained for different configurations of the number of convolutional filters and recursive neurons;
2. In the second experiment, an attempt was made to test to what extent networks consisting only of convolutional layers or only recursive layers will be able to obtain a good classification performance. For this purpose, three models were created and trained for different configurations of the number of convolutional filters and recursive neurons;
3. The next experiment allowed determining the combination of the number of convolutional layer filters and recursive layer neurons, which allowed training the network with the highest performance. For this purpose, the network architecture that achieved the best results in the first experiment was used;
4. The fourth experiment was to check the influence of parameters such as the convolution kernel size and the dropout rate value. The network architecture that obtained the best results in the first experiment and the parameters of convolutional and recursive layers considered the best in the third experiment were used to conduct the investigation;
5. In the fifth experiment, an attempt was made to validate the network predictions for more classes. For this purpose, the data from 40 and 60 users were used, the model was trained, and the results were compared with those obtained with the same architecture and network configuration for 20 classes. The experiment used the model with the highest accuracy from the first experiment and the parameters ensuring the best results in the second, third, and fourth experiments;

### 3.3. Measures

There are different measures that may be utilized for comparing the models; the most popular ones are: accuracy, sensitivity, and precision.

Accuracy is the total number of valid model predictions divided by all predictions. Despite the high index of this value, it may occur that the model does not work correctly—for example, when the data are unevenly distributed and one class has many samples while another has only a few. In such a situation, the network may assign all samples to a more numerous class, which entails high accuracy value.

For every experiment, all three measures were calculated; however, in the presented case, the samples were evenly distributed among classes (users), so the values of precision and recall were more or less the same as accuracy. Therefore, the decision was made to present only accuracy in the following sections.

### 3.4. Experiment 1—Comparing Network Architectures

To solve the problem of user identification based on typing dynamics, the authors in [19] proposed a model consisting of one convolutional layer (Conv1D), two recursive GRU layers, and a fully connected Dense layer. The research described in the above work showed that among tested values, the best results were obtained by the use of the following parameters:

- Kernel size—2;
- Dropout rate—0.5;
- Window size—50.

**Figure 4:**
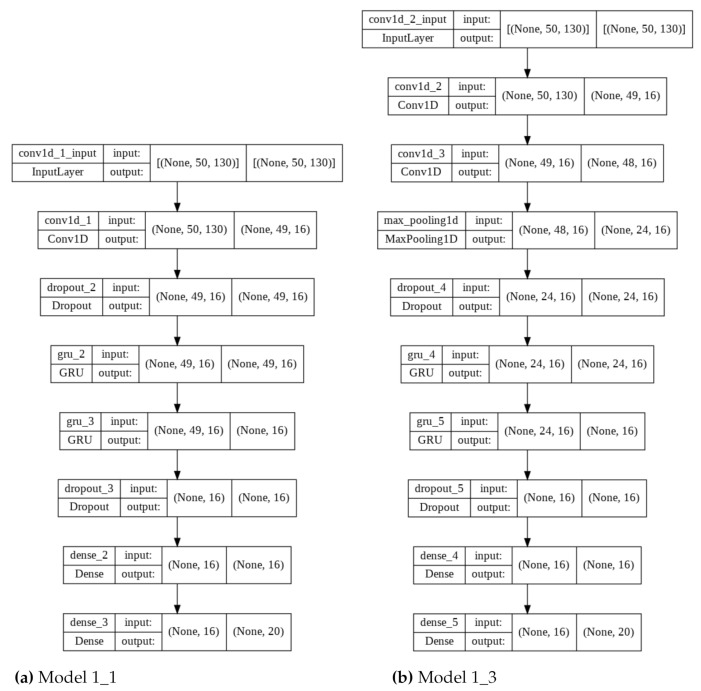
Graphical representations of two models used in Experiment 1 with window size equal to 50, 16 filters, and 16 neurons.

In the experiment, the above-mentioned architecture (Model 1_1) and parameters were used as a reference for the remaining networks. Four additional models were also created with architecture modifications. An additional convolution layer was added in the second model with the same parameters (Model 1_ 2). The next two networks were characterized not only by an additional convolution layer but also MaxPooling1D with a value of 2, added after the Conv1D layer and before (Model 1_3) or after the Dropout layer (Model 1_4). The last model was modified by adding an additional GRU layer (Model 1_5) to the original model. All models were characterized by the same activation functions, in particular: ReLU for convolutional and fully connected layers, and softmax for the last layer. The model architectures are shown in Table 1 and two exemplary graphical representations are shown in Figure 4.

The above models were trained for six combinations of convolutional filter values and recursive network neurons: 16, 32, 64, 128, 256, and 512. Additionally, four window sizes were investigated: 30, 40, 50, and 60.

### 3.5. Experiment 2—Comparing Networks Containing Convolutional or Recursive Layers

The subject of the second experiment was to investigate the performance of models consisting only of convolutional or recursive layers. For this purpose, three networks were created: the first one contained two convolutional layers (Model 2_1), and the next contained two recursive layers: LSTM (Model 2_2) and GRU (Model 2_3). Similarly to Experiment 1, the study was repeated for three numbers of convolution filters and neurons in the recursive network (16, 32, 64) and different window sizes. All models are presented in Table 2.

### 3.6. Experiment 3—Finding the Optimal Number of Filters and Neurons

For the subsequent study, one of the models proposed in the previous experiments was selected, which was characterized by the highest values among the measured indicators (Model 3 from Experiment 1: Model 1_3, described in Table 1). The experiment aimed to find the configuration of the numbers of convolutional filters and neurons of recursive layers, for which the network will achieve the best performance. Parameters such as the size of the convolution kernel and the dropout layer parameter remained unchanged and amounted to 2 and 0.5, respectively.

### 3.7. Experiment 4—Effect of Convolution Kernel Size and Dropout Rate

The subsequent step of the research was an attempt to check the impact of the size of the convolution layer kernel and the value of the dropout rate parameter on the network performance. Once again, Model 1_3 from the first experiment was used for this purpose. The number of convolutional layer filters and recursive layer neurons was 128.

### 3.8. Experiment 5—Wider Set of Classes

Comparing the performance of the created and tuned networks for different numbers of classes (20, 40, and 60) was the next step of the studies. All three tests, for 20, 40, and 60 classes, were performed using the following parameters: 128 convolutional filters, 128 neurons in the recursive network, convolution kernel size equal to 2, dropout rate: 0.5, sample length: 40.

Due to the greater difficulty of the classification task (the higher number of classes), 2000 events instead of 1500 were used for each person from the dataset.

## 4. Results

Data belonging to 20 people after the preprocessing step were used to compare the proposed configurations. For each person, 1500 events containing information about their typing style were loaded, and then, the data were divided into training and test sets in a 3:1 ratio. Each model was trained in 200 iterations with a batch size value of 64. The reported accuracy values are averaged for all classes. The experiments were repeated for four window sizes: 30, 40, 50, and 60. The results for each experiment are presented in relation to the tested architectures.

### 4.1. Experiment 1

Table 3 presents the values of the accuracy for different window sizes. The highest results for a given model configuration are highlighted in bold. The model proposed in [19] achieved the best results only for two configurations: both for window size 30. For samples longer than 30 vectors, the best results were given by Model 1_3, consisting of two convolutional and two recursive layers.

The highest values of the evaluated measure were obtained using Model 1_3 and window size 40. The model’s accuracy was 87%. The number of convolutional filters and neurons of the recursive network was 64.

### 4.2. Experiment 2

The results for the different window sizes are shown in Table 4. The highest results for a given parameter configuration have been marked in bold. The comparison of the measure’s values showed that none of the models returned results better than Model 1_3 from the first experiment.

### 4.3. Experiment 3

The results of the experiment are presented in Table 5. The model with the number of filters equal to 64 and the number of neurons equal to 128 was characterized by the highest accuracy equal to 87%, using window size 40.

### 4.4. Experiment 4

The earlier described network was subjected to training using convolution kernel sizes: 2, 3, 4, 5, and 6. The results are presented in Table 6. The highest accuracy was obtained using the kernel of size 2 (81%). It can also be seen that the network accuracy decreases with the increase of the tested parameter value.

The next part of the study was to check the influence of the dropout rate parameter value. Dropout is a layer that only passes forward some of the weights to prevent overfitting the model during training. Various parameter values were analyzed when investing its influence: from 0.1 to 0.9 with the step equal to 0.1. The obtained results are presented in Table 7.

### 4.5. Experiment 5

As expected, the number of classified classes has an impact on the results. The metrics values are presented in Table 8. Three datasets were tested: the dataset with 20 classes used in the previous experiments and two additional datasets with 40 and 60 classes. The Rank-1 accuracy for the 60-classes dataset is only 69.1%. Still, it is visible that the model produces meaningful results because, for Rank-3, the accuracy rises to 87.3%, which means that in 87% of cases, the correct class is among 5% of the classes with the highest score.

## 5. Discussion

The hyperparameter tuning experiments presented in Section 4 revealed that the obtained results highly depend on the chosen model configuration. Experiment 1 showed that generally, models with more layers could be trained to achieve better performance for the test set. The Model 1_1 taken from the work [19] can be effectively improved with more convolutional (CONV) and recurrent layers (GRU). The best results were achieved by doubling the number of these layers (Model 1_3). The number of filters lower than 64 did not give satisfactory results. It seems that the model needs to calculate more convolutional features to be able to classify samples. For the number of filters from 64 to 256, the results were comparable with the drop of performance for 512 filters. Considering the balance between the complexity of the model and its performance, it seems that 64 or 128 filters is the best option. Similarly, the differences for window sizes 40, 50, and 60 are not significant, and therefore, we conclude that the window size equal to 40 should be sufficient.

Experiment 2 aimed to check which of the layers—convolutional (CONV), LSTM, or GRU—is the best for the task. It did not give the definite answer with the CONV-based model best in four out of 12 trials, LSTM in five out of 12, and GRU in three out of 12. Moreover, all results were worse than for the same window size and number of filters configurations in Experiment 1. It shows that the best solution is mixing CONV and recurrent layers and that the LSTM layers are probably better for the task than GRU layers (but it is not significant).

Further optimization of the best model (Model 1_3) by adjusting the number of filters in CONV and neurons in recurrent layers was done in Experiment 3. It occurred that increasing the number of filters and neurons helps in improving classification results. The best model with 64 filters and 128 neurons for window size 40 gave the accuracy of 87%.

Experiment 4 examined the influence of the kernel size and dropout rate. It occurred that increasing the kernel size does not improve the results—quite the contrary, the results deteriorate. This result is in line with the results presented in [19] for the continuous authentication task. It shows that there are no long-term dependencies between keystroke events that influence identification (and authorization). Only directly neighboring events are meaningful. The way the key is pressed is influenced only by the position of the previous and the next key.

As for the dropout rate, randomly discarding some of the neurons during training significantly improved the ability of the network to learn correctly. Using a low value of the parameter (0.1), in most cases, the network correctly assigns samples to classes for the training set, but for test data, the accuracy is much lower. When using 0.5 as the value of the tested parameter, the difference in losses and accuracies between the training and test sets was much smaller so the model is more generalized and less prone to overfitting with a higher dropout rate. The value of 0.5 is relatively high. It is the evidence that many independent internal representations must be created in the network to correctly identify classes. When the dropout layer removes randomly half of the weights for each sample during training, these independent representations (consisting of the left neurons) are enhanced.

The last Experiment 5 aimed to check how the best model chosen for the dataset with 20 classes behaves for bigger datasets with 40 and 60 classes. Moreover, the amount of data for one class used in this experiment increased from 1500 to 2000 events. The results show that the model is unable to achieve similar performance, but even for 60 classes in 87.3% of cases, the correct result was among 5% of classes with the highest score (Rank-3 accuracy was 87.3%). This result is worse than the identification results presented in the recent paper [24], where the probability that the correct class belongs to 5% of the highest scored classes was for the desktop scenario between 96% and 99% (but from 80.4% to 87.5% for the mobile dataset).

However, it must be emphasized that the results are not directly comparable. The authors of [24] used their network to calculate a similarity between two samples that is convenient for the authorization tasks. To calculate the identification accuracy, they took five samples with unknown classification, calculated their distances with ten samples collected for each user, and then averaged all 50 results. The class with the lowest value was chosen as the identified class. In our case, a model was used that directly classified one sample into one of the N classes.

## 6. Conclusions

The research aimed to develop an artificial neural network model allowing user identification based on typing dynamics for any text. For this purpose, different network architectures were compared using convolutional and recursive layers and their combinations. Then, the influence of hyperparameters such as the number of convolutional filters, the number of neurons of the recursive network, the size of the convolution kernel, the dropout rate, and the window size were investigated.

Considering the complexity of the problem, the accuracy of 88% for identification among 20 users and almost 70% for 60 users seem to be promising. Moreover, Rank-5 accuracies were 98.7% for 20 classes and 90.7% for 60 classes. It was achieved in the end-to-end solution with one model classifying samples directly into classes without any further tuning with thresholds or distances as was done in similar studies. However, further research on this topic is undoubtedly required.

## Figures and Tables

**Table 1:** Layers description for all models used in Experiment 1.

Model 1_1	Model 1_2	Model 1_3	Model 1_4	Model 1_5
Conv1D	Conv1D	Conv1D	Conv1D	Conv1D
Dropout	Conv1D	Conv1D	Conv1D	Dropout
GRU	Dropout	MaxPooling	Dropout	GRU
GRU	GRU	Dropout	MaxPooling	GRU
Dropout	GRU	GRU	GRU	GRU
Dense	Dropout	GRU	GRU	Dropout
Dense	Dense	Dropout	Dropout	Dense
	Dense	Dense	Dense	Dense
		Dense	Dense	

**Table 2:** Layers description for all models used in Experiment 2.

Model 2_1	Model 2_2	Model 2_3
Conv1D	LSTM	GRU
Conv1D	LSTM	GRU
Dropout	Dropout	Dropout
Dense	Dense	Dense
Dense	Dense	Dense

**Table 3:** The accuracy of five models for Experiment 1 using different window sizes and filter numbers.

Window Size	Filters Number	Model 1_1	Model 1_2	Model 1_3	Model 1_4	Model 1_5
30	16	32%	36%	49%	46%	40%
	32	65%	65%	63%	**68**%	63%
	64	**83**%	78%	82%	79%	82%
	128	**84**%	81%	82%	81%	77%
	256	78%	74%	82%	**84**%	81%
40	16	37%	42%	51%	**52**%	48%
	32	65%	57%	**72**%	70%	69%
	64	80%	82%	**87**%	84%	81%
	128	80%	70%	**85**%	81%	80%
	256	77%	73%	**86**%	85%	75%
	512	71%	71%	**84**%	82%	76%
50	16	37%	36%	41%	**50**%	46%
	32	55%	63%	**65**%	62%	64%
	64	73%	74%	**83**%	82%	74%
	128	66%	68%	**79**%	78%	75%
	256	68%	63%	**83**%	82%	66%
	512	60%	67%	**83**%	76%	68%
60	16	36%	34%	43%	**49**%	36%
	32	40%	51%	61%	63%	**66**%
	64	69%	64%	**86**%	78%	72%
	128	53%	46%	**81**%	71%	60%
	256	53%	48%	**70**%	69%	58%
	512	44%	54%	**69**%	65%	66%

**Table 4:** The accuracy for models in Experiment 2 using different window sizes and filter numbers.

Window Size	Filters Number	Model 2_1	Model 2_2	Model 2_3
30	16	**41%**	37%	38%
	32	46%	**51%**	46%
	64	45%	58%	**58%**
40	16	**40%**	36%	36%
	32	33%	**56%**	56%
	64	31%	58%	**59%**
50	16	**40%**	36%	36%
	32	33%	**56%**	56%
	64	31%	58%	**59%**
60	16	**23%**	13%	4%
	32	20%	**55%**	34%
	64	18%	**64%**	52%

**Table 5:** The accuracy of the model in Experiment 3 using a different number of convolutional filters and neurons for recursive layers.

Window	Neurons	Filters Number		
Size	Number	32	64	128
40	16	59%	51%	77%
	32	68%	79%	80%
	64	68%	80%	86%
	128	78%	**87**%	83%
	256	77%	80%	84%
50	16	62%	58%	68%
	32	69%	73%	80%
	64	77%	**84**%	80%
	128	74%	81%	78%
	256	74%	83%	79%
60	16	42%	59%	62%
	32	53%	68%	70%
	64	68%	81%	79%
	128	76%	81%	81%
	256	78%	**83**%	77%

**Table 6:** The accuracy obtained for different convolution kernel sizes.

Kernel Size	2	3	4	5	6
**Accuracy**	81%	79%	75%	70%	61%

**Table 7:** Network accuracy with different *dropout rate* values.

Dropout Rate	0.1	0.2	0.3	0.4	0.5	0.6	0.7	0.8	0.9
**Accuracy**	63%	74%	76%	78%	82%	80%	80%	76%	25%

**Table 8:** The accuracy of prediction for 20, 40, and 60 classes.

Accuracy	Number of Classes		
	20	40	60
Rank-1	88.1%	82.8%	69.1%
Rank-2	96.0%	91.9%	80.9%
Rank-3	97.7%	95.7%	87.3%
Rank-4	98.6%	97.0%	90.6%
Rank-5	98.7%	98.0%	90.7%

## Data Availability

The code and the data is available at https://www.github.com/kasprowski/keystroke2022, accessed on 17 April 2022.

## Abbreviations

The following abbreviations are used in this manuscript:

MDPI	Multidisciplinary Digital Publishing Institute
CNN	Convolutional Neural Network
RNN	Recurrent Neural Network
GRU	Gated Recurrent Unit
LSTM	Long Short-Term Memory
CONV	Convolutional Layer
